# V-ATPase in glioma stem cells: a novel metabolic vulnerability

**DOI:** 10.1186/s13046-025-03280-3

**Published:** 2025-01-17

**Authors:** Alessandra Maria Storaci, Irene Bertolini, Cristina Martelli, Giorgia De Turris, Nadia Mansour, Mariacristina Crosti, Maria Rosaria De Filippo, Luisa Ottobrini, Luca Valenti, Elisa Polledri, Silvia Fustinoni, Manuela Caroli, Claudia Fanizzi, Silvano Bosari, Stefano Ferrero, Giorgia Zadra, Valentina Vaira

**Affiliations:** 1 Department of Pathophysiology and Transplantation, University of Milan, Milan, Italy; 2 Division of Pathology, Fondazione IRCCS Ca’ Granda Ospedale Maggiore Policlinico, Milan, Italy; 3 Molecular and Cellular Oncogenesis Program, Wistar Institute, Philadelphia, PA USA; 4 INGM, Istituto Nazionale Di Genetica Molecolare “Romeo Ed Enrica Invernizzi”, 20122 Milan, Italy; 5 Department of Transfusion Medicine, Precision Medicine Lab, Biological Resource Center, Fondazione IRCCS Ca’ Granda Ospedale Maggiore Policlinico, Milan, Italy; 6 EPIGET-Epidemiology, Epigenetics, and Toxicology Lab, Department of Clinical Sciences and Community Health, University of Milan, 20122 Milan, Italy; 7 Environmental and Industrial Toxicology Unit, Fondazione IRCCS Ca’ Granda Ospedale Maggiore Policlinico, 20122 Milan, Italy; 8 Division of Neurosurgery, Fondazione IRCCS Ca’ Granda Ospedale Maggiore Policlinico, Milan, Italy; 9 Department of Biomedical, Surgical and Dental Sciences, University of Milan, 20122 Milan, Italy; 10 Institute of Molecular Genetics, National Research Council (CNR-IGM), 27100 Pavia, Italy

**Keywords:** Glioma, Glioma stem cell, V-ATPase, Metabolism, Bafilomycin A1

## Abstract

**Background:**

Glioblastoma (GBM) is a lethal brain tumor characterized by the glioma stem cell (GSC) niche. The V-ATPase proton pump has been described as a crucial factor in sustaining GSC viability and tumorigenicity. Here we studied how patients-derived GSCs rely on V-ATPase activity to sustain mitochondrial bioenergetics and cell growth.

**Methods:**

V-ATPase activity in GSC cultures was modulated using Bafilomycin A1 (BafA1) and cell viability and metabolic traits were analyzed using live assays. The GBM patients-derived orthotopic xenografts were used as in vivo models of disease. Cell extracts, proximity-ligation assay and advanced microscopy was used to analyze subcellular presence of proteins. A metabolomic screening was performed using Biocrates p180 kit, whereas transcriptomic analysis was performed using Nanostring panels.

**Results:**

Perturbation of V-ATPase activity reduces GSC growth in vitro and in vivo. In GSC there is a pool of V-ATPase that localize in mitochondria. At the functional level, V-ATPase inhibition in GSC induces ROS production, mitochondrial damage, while hindering mitochondrial oxidative phosphorylation and reducing protein synthesis. This metabolic rewiring is accompanied by a higher glycolytic rate and intracellular lactate accumulation, which is not exploited by GSCs for biosynthetic or survival purposes.

**Conclusions:**

V-ATPase activity in GSC is critical for mitochondrial metabolism and cell growth. Targeting V-ATPase activity may be a novel potential vulnerability for glioblastoma treatment.

**Supplementary Information:**

The online version contains supplementary material available at 10.1186/s13046-025-03280-3.

## Background

Glioblastoma (GBM) is a highly lethal tumor of the central nervous system. It is characterized by a high degree of intra-tumor heterogeneity, in terms of histological and molecular features [1]. At the molecular level, primary GBM is characterized by wild-type IDH enzymes and EGFR amplification [2]. The elevated lethality of glioblastoma and poor response to therapeutics are due to the presence of the glioma stem cell (GSC) niche, which is favored by a hypoxic microenvironment [3]. GSCs play a crucial role in tumor initiation, progression, therapy resistance, and disease recurrence due to their ability to self-renew and differentiate into various cell types. The metabolism of GSCs is quite differ from that of normal brain cells and cells of the tumor bulk [4]. Indeed, GSCs show metabolic plasticity, enabling them to favor glycolysis or OxPhos as source of energy, according to environmental conditions. This metabolic plasticity is crucial for adapting to nutritional and oxygen changes within the tumor microenvironment (TME), allowing them to adapt to and/or compensate for an unfavorable TME and contributes to their superior resistance to therapeutics. [4, 5]. Therefore, the identification of potential novel targets to halt GSCs survival has become a priority in the medical setting. Targeting the metabolic vulnerabilities of GSCs has proven a promising approach to eliminate GSCs [4].

Alterations in cellular metabolism and pH gradient have been recognized as hallmarks of cancer, including GBM. Hypoxia and hypoxia-inducible factors (HIFs) play a crucial role in promoting metabolic reprogramming in cancer cells as well as in promoting stemness and therapy resistance [3, 6].

The vacuolar proton pump V-ATPase is one of the main regulators of intra/extra-cellular space acidification and nutrient sensing. V-ATPase is a multi-subunit protein responsible for protein trafficking, endo/lysosomal activity, mTORC1-directed cell growth, and autophagy [7, 8]. In physiologic conditions, V-ATPase activity modulates neuron synaptic activity through the loading of neurotransmitters into synaptic vesicles [9]. Recent papers have showed that the expression of V-ATPase subunits is deregulated in tumor cells and is not restricted to lysosomes only [10–14]. Changes in V-ATPase subunits expression, localization and mutations are closely associated with cancer progression and invasion [11, 15]. Furthermore, alterations in V-ATPase activity can impair chemotherapeutics uptake and efficiency through the modulation of extracellular pH [16]. Because of the pleiotropic effects of V-ATPase activity, this pump represents an attractive therapeutic target in translational oncology [15, 17, 18].

We have previously showed that the overexpression of the catalytic subunit V1G1 correlates with increased GSCs viability, clonogenicity and invasiveness. These features were significantly reduced by impairing V-ATPase activity with Bafilomycin A1 (BafA1) treatment. During GSCs differentiation, we also observed significant reduction of V1G1 expression [19]. Finally, we demonstrated that V-ATPase activity in GSCs participates in reprogramming the brain microenvironment toward a pro-tumorigenic state through extracellular vesicles [20, 21]. Specifically, extracellular vesicles from GSCs with high V-ATPase V1G1 subunit expression induce ERK1/2, Notch and PI3K/mTOR signals in non-neoplastic recipient brain cells, thus increasing their proliferative activity, survival, and motility [20, 21].

The PI3K-AKT-mTOR signaling is a key oncogenic pathway in GBM, where it regulates tumor metabolism, translation, cell growth and autophagy [22, 23]. The latter is altered in human primary GBM GSCs and in a Drosophila model of glioma with high V-ATPase expression. Indeed, restoration of the autophagic flux through the downregulation of V-ATPase subunits or Target of rapamycin complex 1 (TORC1) resulted in the inhibition of Akt signaling and of glial cell overgrowth [24].

While the critical role of V-ATPase in -GBM is now established, how this pump contributes to GSCs viability and growth is still unknown. Thus, understanding the underpinning pro-tumorigenic metabolic mechanisms and pathways regulated by V-ATPase is crucial to propose new therapeutic opportunities. In this study we characterize the role of V-ATPase in regulating bioenergetics in GSCs and we provide novel insights into the mechanisms behind BafA1-induced cell death.

## Methods

The following methods are described in the Supplementary Information file: Total and mitochondrial protein isolation and quantification; Immunoblotting and protein array; Standard, calibration and quality control solutions; LC–MS/MS analysis; list of antibodies used.

### Patients’ samples, GSCs culture and pharmacological treatment

Tumor samples from GBM patients’ (listed in Suppl. Table S1) were obtained from the Neurosurgery Unit of Fondazione IRCCS Ca’ Granda Ospedale Maggiore Policlinico. The study was approved by a local Ethic Committee (IRB#275/2013) and all patients signed an informed consent. GBM tissues were dissociated using both enzymatic and mechanical methods (Tumor dissociation kit, Miltenyi Biotech). GBM-derived neurospheres enriched in GSCs [19], were cultured in Neurocult medium supplemented with EGF and FGF (STEMCELL Technologies) as previously described [19]. Experiments were performed in technical triplicate using primary GSCs derived from different GBM patients (described in Suppl. Table S1). GSCs were subjected to the following treatments: BafilomycinA1 (5 or 20 nM for 24 and 48 h; sc-201550, Santa Cruz Biotechnology); ROS inhibitor MitoTempo (100 µM for 24 h; SML0737, Sigma-Aldrich); D-glucose-^13^C_6_ (10 mM for 6 h; 389,374, Sigma-Aldrich).

### Fluorescent assays

For apoptosis detection, GSCs were seeded in a 96 well plate and stained with Annexin V (cat. 556,419, BD Bioscience) for 15 min at 37 °C. Images were captured using a Nikon time-lapse microscope (Eclipse Ti-E Nikon) at 5 × of magnification. Lysosomal activity was analyzed by incubating GSCs with DQ-BSA (D12051 Thermo Fisher Scientific) for 24 h, then GSCs were cytospinned, fixed in 4% Paraformaldehyde (PFA) and mounted using the ProLong gold antifade reagent (Thermo Fisher Scientific).

For Proximity Ligation Assay (Duolink PLA – Sigma) and immunofluorescence (IF) GSCs were cytospinned, fixed in 4% PFA, permeabilized in PBS-Triton 0.5% and incubated 60 min at 37 °C with Duolink Blocking Solution for PLA and in BSA 10% for IF. Cells were then incubated overnight at 4 °C with the following primary antibodies: anti-V-ATPase G1 and anti-Tomm20 (for PLA and IF), anti-V-ATPase G1 and anti-Lamp1 (for PLA). For both the procedures single primary antibodies staining was performed as background controls. Antibodies dilution is reported in Suppl. Table S2.

For PLA, PLUS and MINUS probes, ligation and amplification were performed following the manufacturer instructions. Slides were then mounted using Duolink in Situ Mounting with DAPI. For IF, cells were stained with fluorescent secondary antibody (Suppl. Table S2), and nuclei were stained with Hoechst 3342 (1:1000, Cell Signaling). Slides were mounted with coverslip and ProLong Gold antifade (P36930, Thermo Fisher Scientific). Images were acquired with Leica SP8 Confocal Microscope (Leica Microsystems), z stack 0.46 μm, at a magnification of 63x.

Protein synthesis was evaluated incubating GSCs with the Click-it Plus OPP assay (20 μM; cat. C10456, Thermo Fisher Scientifics) for 1 h. Then, cells were rinsed twice and cytospinned at 300 rpm for 3 min, fixed with PFA 4% and permeabilized with PBS-Triton 0.5%. GSCs were then processed following the manufacturer instruction. Images were acquired using a Leica SP8 Confocal Microscope, z stack 0.46 μm, at a magnification of 63x. All fluorescent images were quantified using Fiji ImageJ software (https://imagej.net), with the following parameters: for AnnexinV and protein synthesis, mean fluorescence intensity (MFI) of positive spots was measured, whereas for DQ-BSA and PLA assay total positive spot area was calculated.

For colocalization experiments and 3D reconstruction, images were deconvolved using Hyugens software and 3D images were reconstructed using the Leica software. The colocalization was measured as the percentage of V1G1 signal that colocalizes with Tomm20 signal.

### Cell staining and flow cytometry analysis

GSCs spheres were dissociated to single cell, filtered to avoid aggregates and washed in PBS. For the evaluation of mitochondria ROS levels, cells were stained with MitoSoX Red (1:1000 M36008, Molecular Probes) in HBSS for 10 min at 37 °C. To assess mitochondria depolarization, cells were stained in growth medium at 37 °C with TMRE (50 nM; T669, Thermo Fisher Scientific) for 10 min or with JC1 (MBS258016, MyBioSource) for 25 min. For cell cycle analysis, cells were fixed with ethanol 100% overnight at 4 °C, washed and stained with a mix of propidium and RNAse (550,825, BD Bioscience) for 15 min at 4 °C. Samples were acquired using FACSCanto II (BD Biosciences), data were subsequently analyzed with FlowJo v.10 software by Dean-Jett-Fox model.

### Real-time cell metabolic analysis

Metabolic features of GSCs were analyzed using the Seahorse XFe24 Analyzer, and the XF Real-Time ATP Rate Assay Kit, XF Cell Mito Stress Test Kit and XF Glucose/Pyruvate Oxidation stress test kit (all from Agilent Technologies). GSCs were grown in NC medium and treated for 48 h with BafA1. After 48 h cells were collected, washed (Seahorse XF DMEM medium, pH 7.4 w/o Phenol Red, Glucose 10 mM, Pyruvate 1 mM, L-Glutamine 2 M) and 100 μl/well of GSCs suspension was seeded in a XF24 islet capture plate (Mito Stress Test and for Glucose/Pyruvate Oxidation stress test) or in a XF24 V7 plate coated with poly-L-lysine (P4832 Sigma) (ATP rate assay). Medium was added to a final volume of 500 μl. Plates were incubated in a 37 °C non-CO2 incubator for 45 min and then loaded in the instrument. Cartridge ports were loaded with Oligomycin (Cf:4 µM), FCCP (Cf: 4 µM), Rotenone + Antimycin A (Cf:1.5 µM), UK5099 (Cf:2 µM) according to manual instructions. At the end of the experiment, pictures of each well were taken, and spheres total area was calculated by Fiji ImageJ software. Area values were used to normalized metabolic assays. For the ATP Rate Assay total proteins were extracted from GSCs, quantified and used to normalize data. Data were analyzed using the Seahorse Analytics (https://seahorseanalytics.agilent.com). Only GSCs-containing wells with a O_2_ pressure equal to 152 ± 10 mmHg and an OCR value of at least 20 pmol/min were included in the analyzes.

### RNA purification and Nanostring analysis

Total RNA was purified using the MasterPure RNA purification Kit (Epicentre, Illumina). RNA quality and concentration was evaluated by RNA ScreenTape Analysis on a 4200 TapeStation System (Agilent). Gene expression was evaluated using the Cancer Metabolism or the Metabolic Pathways Panel on the nCounter Flex Analysis System (Nanostring). Data analysis was performed using R software (v.4.3.1) and the NanoStringClustR package. For data normalization a modified version of the “multi_norm” function was used. Data were loess normalized, and genes were considered expressed if their expression was greater than or equal to two-times the standard deviation of the negative controls in at least two samples. Genes were considered significantly differentially expressed if they had an adj pvalue (FDR) < 0.05 and a log (FC) >|1|. Genes clusters were identified using the *k*-means function and the silhouette coefficient; then, for each *k*-cluster we performed gene set enrichment analysis using the pathfindR package considering the HALLMARK dataset as reference.

### Targeted metabolomics

GSCs lysates were analyzed using the AbsoluteIDQ® p180 kit (Biocrates Life Sciences AG, Innsbruck, Austria) and LC–MS/MS. To this end, cell pellets were resuspended in cold EtOH 85% (85:15 PBS) followed by two cycles of sonication and freezing and a final centrifugation at 18000 g for 10 min at 4° C. Samples were analyzed with a high-pressure liquid chromatograph Agilent 1260 (Agilent Technologies) coupled with a hybrid triple quadrupole/linear ion trap mass spectrometer (QTRAP 5500; Sciex, Milan Italy) with an electrospray ionization source.

The Analyst® software (version 1.6.3; Sciex) was used to prepare the sequence of analyzes, the MultiQuant™ software (version 3.0.8664.0; Sciex) was used for the integration of chromatographic peaks. Metabolites levels were normalized on the total protein content.

### ^13^C-glucose flux analysis

GSCs were incubated with 10 mM of ^13^C-labeled D-glucose (for 6 h in DMEM medium supplemented with 1 mM pyruvate and 2 mM Glutamine. After the incubation, cell culture medium was collected and centrifuged at 300 g for 5 min to remove cell remains. Supernatant (50 µL) was added with 200 µL MeOH and 5 µL of the internal standard (IS) solution; the sample was mixed with a vortex and then centrifuged at 11,000 g for 10 min, to allow protein precipitation. An aliquot of 10 µL was then injected in LC–MS/MS system for analysis. Cell pellet was washed twice with PBS and resuspended in 80% cold methanol. Samples were vortexed and centrifuged at 12000 g 10 min 4 °C. The obtained extract (100 µL) was evaporated under a gentle stream of nitrogen with the heating block set at 45 °C, reconstituted with 50 µl of MeOH, vigorously mixed with a vortex, and then transferred into an insert. An aliquot of 10 µL was injected in LC–MS/MS system. Samples were analyzed on a Surveyor high performance liquid chromatography system (Thermo Scientific, Rodano, Italy) equipped with a Raptor Polar X, 50 × 2.1 mm, 2.7 µm particle size (Restek, Milan, Italy). The liquid chromatography instrument was interfaced with a triple quadrupole mass spectrometer equipped with a hot-electrospray ionization source (TSQ Quantum Access with H-ESI; Thermo Scientific). Total protein content was used for data normalization.

### Animal experiments

Animal experiments were carried out in compliance with the institutional guidelines for the care and use of experimental animals (European Directive 2010/63/UE and the Italian law 26/2014), authorized by the Italian Ministry of Health and approved by the Animal Use and Care Committee of the University of Milan. Female NOD/SCID mice (n = 10; 7–8 weeks of age, Envigo, Huntingdon, UK) were kept in the appropriate cages in an environment of 23 ± 1 °C and 50 ± 5% humidity, with a 12 h light/dark cycle and fed ad libitum. The orthotopic murine model was obtained by stereotaxic injection (coordinates: 1.5 mm lateral to the bregma, 0 mm behind and 3·0 mm ventral to the dura) of 1 × 10^5 patients derived GSCs stably transduced with a luciferase construct in 2 μl of PBS as described [25]. The effect of V-ATPase activity inhibition on GSCs growth was investigated in mice inoculated with GSC treated with BafA1. Following surgery, mice were monitored for recovery until complete awakening. For bioluminescence imaging, mice were anaesthetized with mixture of tiletamine/zolazepam (40 mg/kg, Zoletil) and xylazine (8 mg/kg, Xilor), and then intraperitoneally injected with 150 mg/kg of luciferin. Bioluminescence signal (BL) was acquired weekly with the IVIS SPECTRUM/CT instrument (Perkin Elmer), using an acquisition time of 5 min, medium binning, f/stop = 1, no filter, field of view 13 × 13 cm. All images were scaled and quantified by applying Regions of Interest (ROI) on BL signals using the Living Image Software. Data were expressed as BL counts. At day 144, all mice were sacrificed and brain was collected, formalin fixed, and paraffin embedded. For morphological and histological examination, 4 μm sections were cut and either stained with hematoxylin and eosin (H&E) or with a STEM121, Ki67, and Ser240/244 phosphorylated S6 (pS6) primary antibody (Suppl. Table S2) as described [25].

## Results 

### V-ATPase inhibition reduces GBM growth in vivo and in vitro

We previously showed that different conformations of the V-ATPase pump are expressed in low-grade glioma respect to GBM and that the pump conformation affects glioma growth in vivo. [25]. High expression of the catalytic V-ATPase G1 subunit is significantly associated with most aggressive gliomas. [19]. Therefore, we started this study inhibiting V-ATPase activity using Bafilomycin A1 (BafA1), a macrolide molecule that binds to the V0 domain of the pump thus inhibiting its rotation and proton translocation [26].

**Fig. 1:**
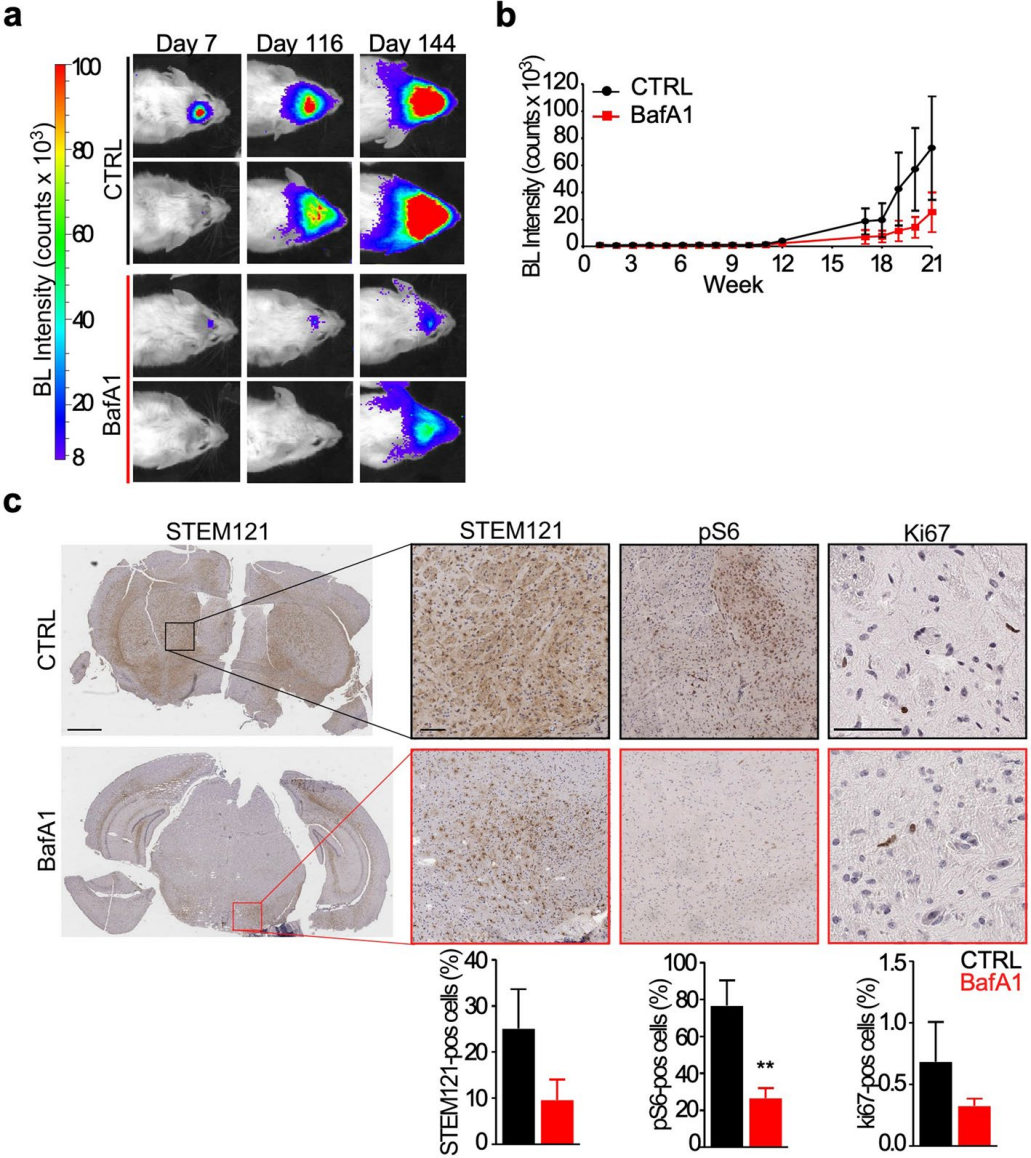
V-ATPase inhibition in GSCs decreases glioma growth in vivo. **a, b** Luciferase-transduced GSCs treated with vehicle or 5 nM BafA1 for 24 h were intracranially injected in nude mice and glioma growth in vivo was evaluated by luciferase emission up to 144 days (BL Intensity; **a,b**). At sacrifice brains were collected and formalin-fixed and paraffine-embedded for morpho-histological examination. Then, a section was stained with STEM121, phosphorylated S6 at Serine 240/244 (pS6) and ki67 antibodies (brown color; **c**) to visualize respectively glioma cell infiltration in the brain parenchyma, cell growth and proliferation. STEM121, pS6 and ki67 staining was quantified using Image Scope (Leica) (**j**). Scale bar 1 mm left panel, 100 μm zoomed inset. Representative images are shown for A and C panels. **, *p* = 0.003 by Mann–Whitney U test

At the cytostatic concentration of 5 nM BafA1 (Suppl. Figure 1a,b), the pump activity in lysosomes was completely inhibited (Suppl. Figure 1c,d) and cell cycle progression was arrested in S phase (Suppl. Figure 1e,f). In the orthotopic mouse model of GBM, [25], treatment of GSCs with BafA1 was sufficient to slow tumor growth (Fig. 1a,b), and to decrease tumor cell invasion (STEM121), proliferation (Ki67) and growth (p-S6) (Fig. 1c).

BafA1 is a well-known inhibitor of the last stages of autophagy [24, 27], a pathway deeply connected with lysosomal function that could promote cell death [28]. Therefore, we analyzed whether autophagy was affected after V-ATPase block. In BafA1-treated GSCs the autophagic markers LC3B-II and p62 showed only a marginal accumulation over time (Suppl. Figure 2a,b), while no difference in autophagosome could be detected in BafA1-treated GSC compared to controls (Suppl. Figure 2c), supporting the previous observations about autophagy impairment upstream of autophagosome formation in GSC with elevated expression of V-ATPase [24].

In keeping with this, the lysosomal compartment was not affected by the V-ATPase block, given that Lamp1 expression and the number of lysosomes was unaffected by BafA1 (Suppl. Figure 2b,e respectively), and the major transcription regulator or the autophagy-lysosomal pathway TFEB was not detected in GSC nuclei (Suppl. Figure 2f). Therefore, this information supports the idea that autophagy is not a pathway key for GSC bioenergetic requirements.

On the contrary, the master energy sensor AMP-activated protein kinase (AMPK) was activated after BafA1 treatment (Suppl. Figure 3a-e) while the activity of the kinase p70S6K (Suppl. Figure 3b,c), the ribosomal protein S6 (Suppl. Figure 3f,g) and of eukaryotic initiation factor 2 (eI2F) alpha subunit (Suppl. Figure 3d,e) was inhibited suggesting that the mTORC1 signaling was inactivated by BafA1 treatment and global translation was compromised (Suppl. Figure 3a).

These data indicate that the inhibition of V-ATPase activity induces a state of energy deficit in primary GSCs without the involvement of autophagy. This metabolic change is crucial for GSCs growth in vivo*.*

### V-ATPase modulates mitochondria homeostasis in GSCs

**Fig. 2:**
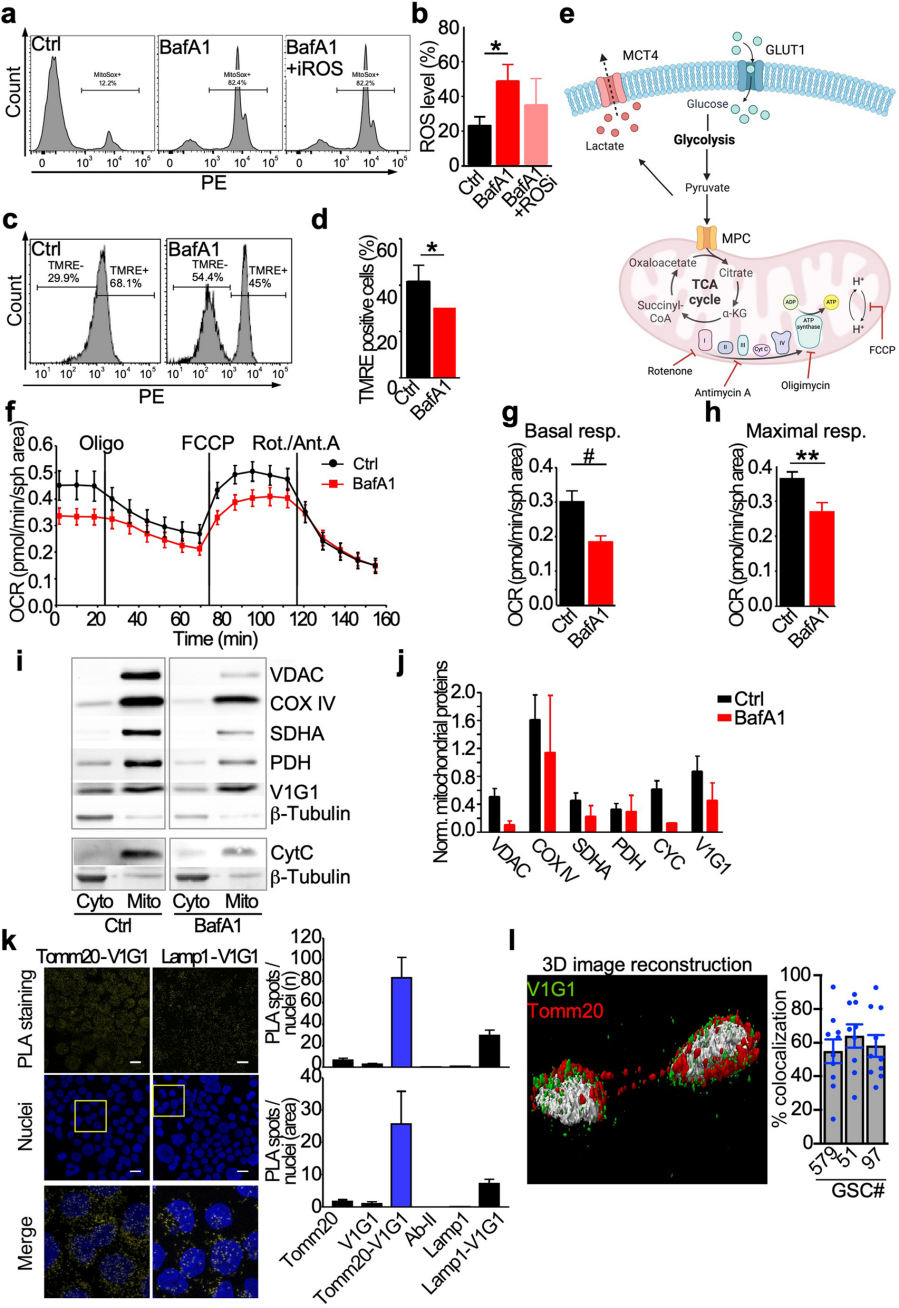
A mitochondrial pool of V-ATPase is present in GSC and V-ATPase targeting perturbs mitochondria homeostasis. **a-d** GSCs were incubated with vehicle (Ctrl) or 5 nM BafA1 for 48 h and then ROS production (**a,b**) or a mitochondrial membrane potential assay (TMRE; **c,d**) were performed. *, *p* = 0.03 by Mann–Whitney U test. **e–h** The mitochondrial function (**e,f**) was evaluated live in GSCs incubated with vehicle (Ctrl) or 5 nM BafA1 for 48 h as the oxygen consumption rate (OCR) using selective uncouplers of the electron transport chain (oligomycin, FCCP, and rotenone-antimycin A). The basal (**g**) and maximal (**h**) respiratory capacity was then measured in GSCs. **, *p* = 0.005; #, *p* = 0.008 by Mann–Whitney U test. **i,j** Mitochondrial proteins were analyzed by immunoblot (**i**) separately in cytoplasmic (Cyto) and mitochondrial (Mito) extracts and quantified by densitometric analysis (**j**) in GSCs treated as in **a**. β-tubulin was used to verify cytoplasmic contamination. **k** A proximity ligation assay (PLA) was performed with antibodies recognizing mitochondria (Tomm20) and V-ATPase G1 (V1G1), or lysosomes (Lamp1) and V-ATPase G1. Nuclei were stained with Hoechst 33,342. *Left*, representative images; scale bar, 20 µm. *Right*, quantification of PLA spots per nuclei. Double Tomm20-V1G1 and Lamp1-V1G1 staining were compared to single antibody staining. See Supplementary Fig. 3C for controls. **l** Colocalization of V-ATPase G1 (V1G1) with mitochondria (Tomm20) in GSCs was analyzed by confocal microscopy followed by 3D deconvolution. *Left*, representative image; scale bar, 20 µm. *Right*, quantification of colocalization was performed with Leica software. Bars, mean with SEM

Next, we better characterize GSCs bioenergetics following V-ATPase inhibition, to elucidate the contribution of the proton pump to GSCs viability. BafA1 treatment in GSCs increased reactive oxygen species (ROS) levels (Fig. 2a,b), and induced mitochondria depolarization (Fig. 2c,d and Suppl. Figure 4a). ROS levels were not reverted to basal conditions after incubation with ROS inhibitor (Fig. 2a,b), suggesting that the inhibition of V-ATPase activity causes irreversible mitochondrial damage. To corroborate this finding, we performed ultrastructural analysis of GSCs at baseline or after BafA1 treatment. Despite the total number of mitochondria was not affected by V-ATPase activity, BafA1 treatment increased damaged organelles while diminishing the number of healthy ones (Suppl. Figure 4b-d). Using a live cell metabolic assay to dynamically measure mitochondrial function (Fig. 2e), we found that V-ATPase impairment decreases basal (Fig. 2f,g) and maximal (Fig. 2f,h) respiration in GSCs cultures. In contrast, the non-mitochondrial respiration was unaffected by BafA1 treatment (Suppl. Figure 4e).

Analysis in purified mitochondrial extracts of enzymes from the matrix and the outer, and inner membrane showed that their expression was reduced after BafA1 treatment (Fig. 2i,j), without modulation of mitophagy (Suppl. Figure 5a,b). Further, we observed V-ATPase G1 localization at the organelle membrane (Fig. 2 i,j).

While a connection between the proton pump and mitochondria, the cell powerhouse, has already been shown in C. Elegans [29] and in a zebrafish model of deafness [30], no evidence in mammalian systems has been reported before.

To validate our novel finding, we analyzed V-ATPase G1 subcellular localization in GSCs using a proximity ligation assay (PLA) and confocal microscopy followed by 3D deconvolution. Both PLA (Fig. 2k and Suppl. Figure 5c) and confocal microscopy (Fig. 2l) showed that the V-ATPase G1 subunit colocalizes with Tomm20, confirming its presence on GSCs mitochondria stained with mitochondrial marker Tomm20. We also analyzed and detected the V-ATPase subunit in lysosomes by PLA (Fig. 2k; Lamp1-positive spots) as expected [7], while no interaction was appreciated between mitochondria and lysosomes (Suppl. Figure 5d).

These data suggest the involvement of mitochondrial V-ATPase in regulating GSCs bioenergetics, resulting in changes in mitochondria homeostasis.

### Mitochondrial metabolism is hijacked by inhibition of V-ATPase activity

**Fig. 3:**
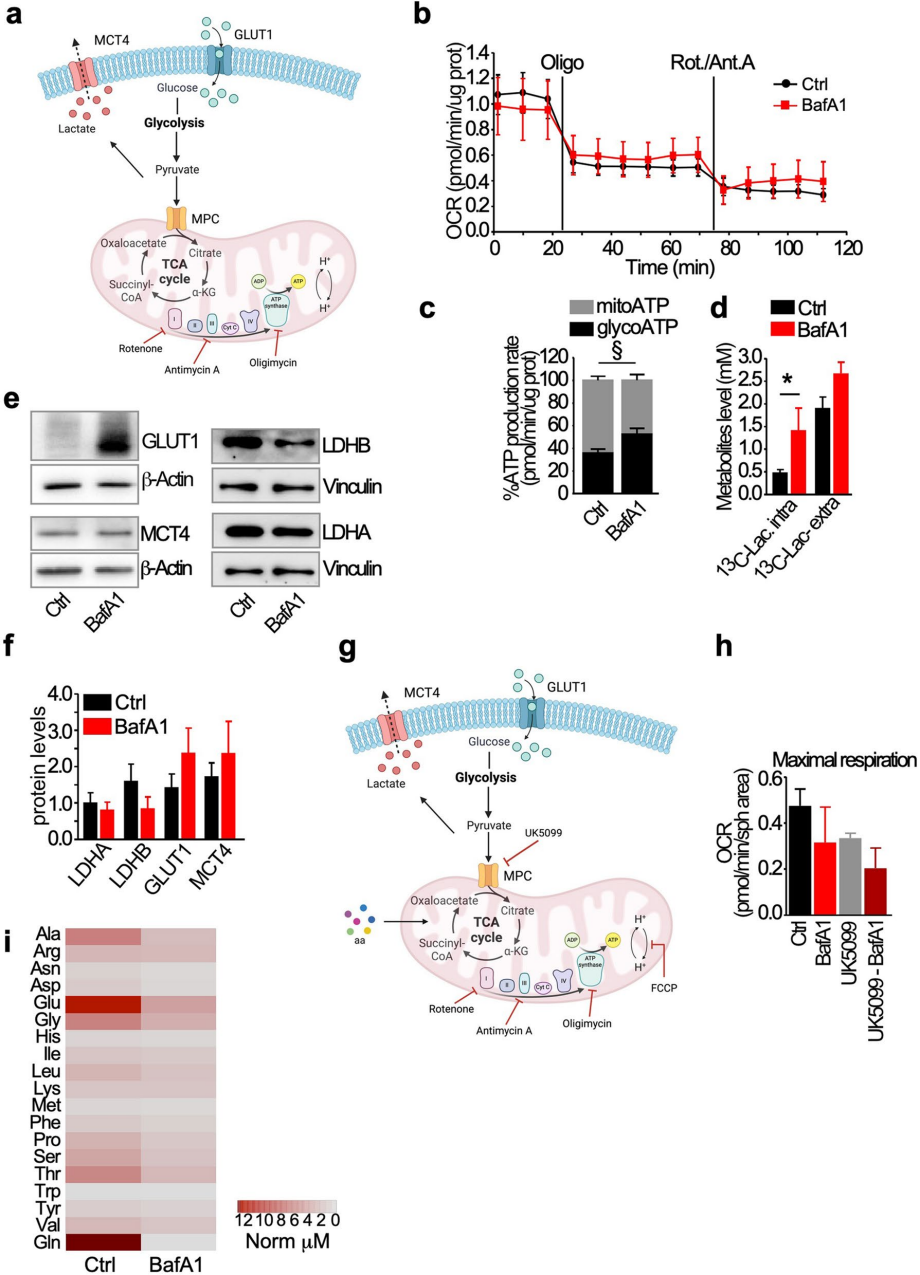
Inhibition of V-ATPase activity reprograms GSCs metabolism. **a-c** A live ATP-rate assay was performed to simultaneously detect energy production from glycolysis and mitochondria in GSCs treated with vehicle (Ctrl) or with 5 nM BafA1 for 48 h. The oxygen consumption rate (OCR; **b**) was measured after oligomycin and rotenone-antimycin A injection and ATP produced by mitochondrial respiration (mitoATP) or by glycolysis (glycoATP) was measured and expressed as percentage of the total ATP (**c**). §, *p* = 0.012 by Mann–Whitney U test. D) GSCs cultures were incubated with ^13^C-glucose and the treated as in A. Lactate production was assessed by a Surveyor high performance liquid chromatography system. *, *p* = 0.026 by Mann–Whitney U test. **e,f** GSCs were treated as in A, then the expression of the indicated metabolic enzymes was analyzed by immunoblot (**e**) and quantified by densitometric analysis (**f**). β-Actin was a loading control. **g,h** GSCs were treated for 48 h with vehicle (Ctrl), 5 nM BafA1, the pyruvate transporter inhibitor UK5099 or the combination of BafA1 and UK5099 (**g**). Then, mitochondria reliance on pyruvate was assessed measuring the OCR and the maximal respiration capacity was determined (**h**). **i** Targeted metabolic profiling was performed in Ctrl- or BafA1-GSCs cell extracts. The heatmap show significantly decreased levels of amino acids in BafA1-treated cultures (for complete list see Suppl. Table S3)

To get further insights into the reliance of GSCs on V-ATPase activity to preserve viability and bioenergetics, we evaluated basal ATP production rates from mitochondrial respiration and glycolysis in living GSCs (Fig. 3a). Following BafA1 treatment, GSCs showed decrease in PDH protein expression (Fig. 2i,j) and mitochondrial ATP production (Fig. 3b,c) and significant increase in glycolysis-derived ATP production as well as lactate intra-cellular accumulation (Fig. 3b-d), suggesting a downregulation of TCA/ oxidative phosphorylation (OxPhos) metabolism and a compensatory enhancement of glycolytic metabolism. In keeping with this, we observed increase of GLUT1 levels, modest decrease of LDHB. No modulation of LDHA and MCT4 (Fig. 3e,f) were observed, indicating that the increased glycolytic rate/lactate accumulation is most likely sustained by increased glucose uptake from the extracellular space. Combination of BafA1 and UK5099, a selective inhibitor of pyruvate import into the mitochondria, induces a further decrease maximal respiration, confirming the impairment of using pyruvate as energetic source under BafA1 treatment (Fig. 3g,h).

In line with this, increased pyruvate reduction to lactate and reduced pyruvate entry into mitochondria affects the levels of pyruvate-derived amino acids, including alanine, aspartate, threonine, and mostly glutamine/glutamate (Fig. 3i, Suppl. Table S3). Equally possible is that their reduction reflects also their use as compensatory energetic/biosynthetic sources in the TCA. Indeed, GSCs have been previously shown to rely on Gln for survival and for maintaining ATP production, cell growth and survival [31].

As lactate acts as signaling molecule to activate transcription of genes involved in mitochondria biosynthesis and pro-survival factors in normal astrocytes and in neuroblastoma cells, we assessed whether this might occur in our system as well [32, 33].

In contrast to previous reports, gene expression data showed that NFR1, a transcription factor involved in mitochondrial DNA replication, and several cell-cycle related genes were decreased in BafA1-treted GSCs (Suppl. Figure 6a,b), suggesting that increased intra-cellular lactate levels cannot compensate for BafA1-mediated mitochondrial metabolism dysregulation.

### Bafilomycin A1-mediated metabolic reprogramming of GSCs

**Fig. 4:**
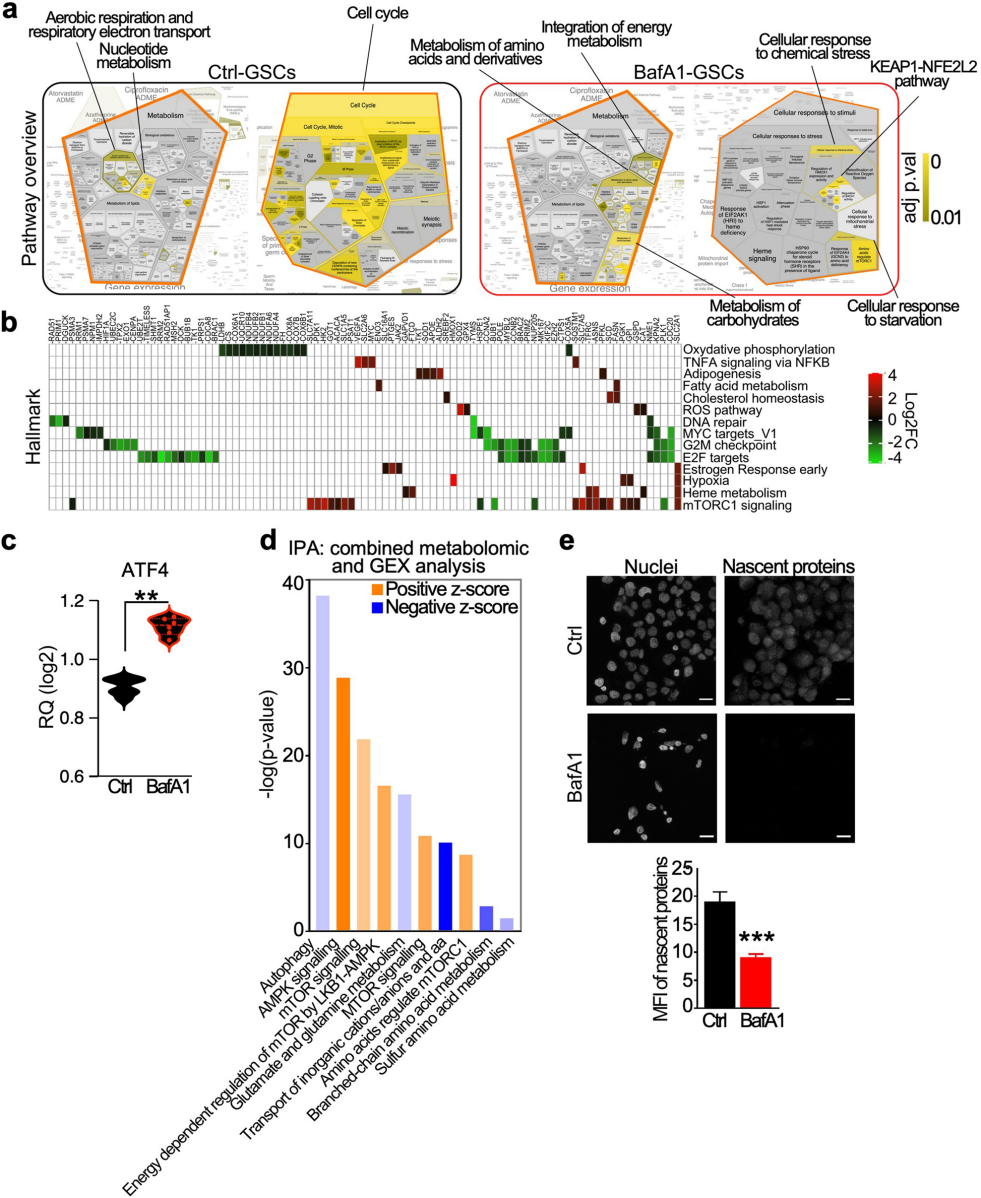
Molecular insights into BafA1-mediated metabolic rewiring of GSCs. **a** A metabolic gene expression panel (n = 748 genes; see also Suppl. Table S4) was analyzed in GSCs treated with vehicle (Ctrl) or 5 nM Bafa1 for 48 h. List of genes whose expression was significantly up (adj p value < 0.05 and log2FC > 0.5) in Ctrl-GSCs or in BafA1-GSCs were separately imported in Reactome web-tool and the pathway analysis was performed with voronoi pathway visualization. The most relevant activated signaling in Ctrl- or BafA1-GSCs are shown. For the complete voronoi charts please refer to Suppl. Figure 7. **b** Transcripts belonging to the indicated hallmark and up (red) or down-modulated (green) in BafA1-treated GSCs are shown (see also Suppl. Table S5). **c** The expression of the activating transcription factor 4 (ATF4) was analyzed in Ctrl- and BafA1-treated GSCs. Data are showed as violin plots and each dot is a sample. **, *p* = 0.002 by Mann–Whitney U test. **d** Ingenuity pathway analysis was performed integrating gene expression (GEX) and metabolic data. Pathway predicted as activated or inhibited (positive and negative z-score, respectively) in BafA1-GSCs are shown (see also Suppl. Table S7). **f** Protein de novo synthesis was evaluated in GSCs incubated for 24 h with vehicle (Ctrl) or 5 nm BafA1 using fluorescence microscopy. The mean fluorescence intensity (MFI) was measured and quantified by Image J software (bottom panel). Scale bar, 20 µm. ***, *p* < 0.0001 by Mann–Whitney U test

To further support results from metabolomics/metabolic assays, we analyzed the expression levels of metabolic-related genes in control and BafA1-treated GSCs (Suppl. Table S4). In line with results from metabolomics/metabolic assay, transcriptomics data showed that GSCs are characterized by proliferative signaling and OxPhos as main metabolic pathway (Fig. 4a and Suppl. Figure 7a). Treatment of GSCs with BafA1 shut-downs OxPhos while activates glucose metabolism and induces of amino-acids stress responses (mTORC1) (Fig. 4a and Suppl. Figure 7b). Hallmark analysis of K-means clusters (Suppl. Figure 8a-c and Supplementary Table S5) confirmed down regulation in BafA1-treated GSCs of key genes involved in oxidative phosphorylation, such as NADH dehydrogenase and Cytochrome C Oxidase subunits, and cell cycle progression, including CDC20, POLE, BUB1 as well as the Polo Like Kinase 1 (PLK1), which has been reported as crucial for GSCs stemness and viability (Fig. 4b) [34]. In contrast, BafA1-treated GSCs upregulated antioxidant genes (ROS hallmark), transcripts involved in fatty acid de novo synthesis (FASN, ACACA, SCD). In regards of the mTORC1 pathway, BafA1-treated GSCs up-regulated factors involved in amino acids and glucose transport (Solute Carrier Family members) (Suppl. Figure 8d), glucose metabolism (HK2, PFKL, GPI, GSPD), as well as anabolic enzymes (GOT1, ASNS) (Fig. 4b, Suppl. Figure 8a-c and Suppl. Table S6).

These data depict a scenario whereby BafA1-GSCs as cells are facing hindered proliferation and an energetic/biosynthetic crisis, demanding sources from the extracellular milieu antioxidant enzymes to dampen increased ROS [31].

**Fig. 5:**
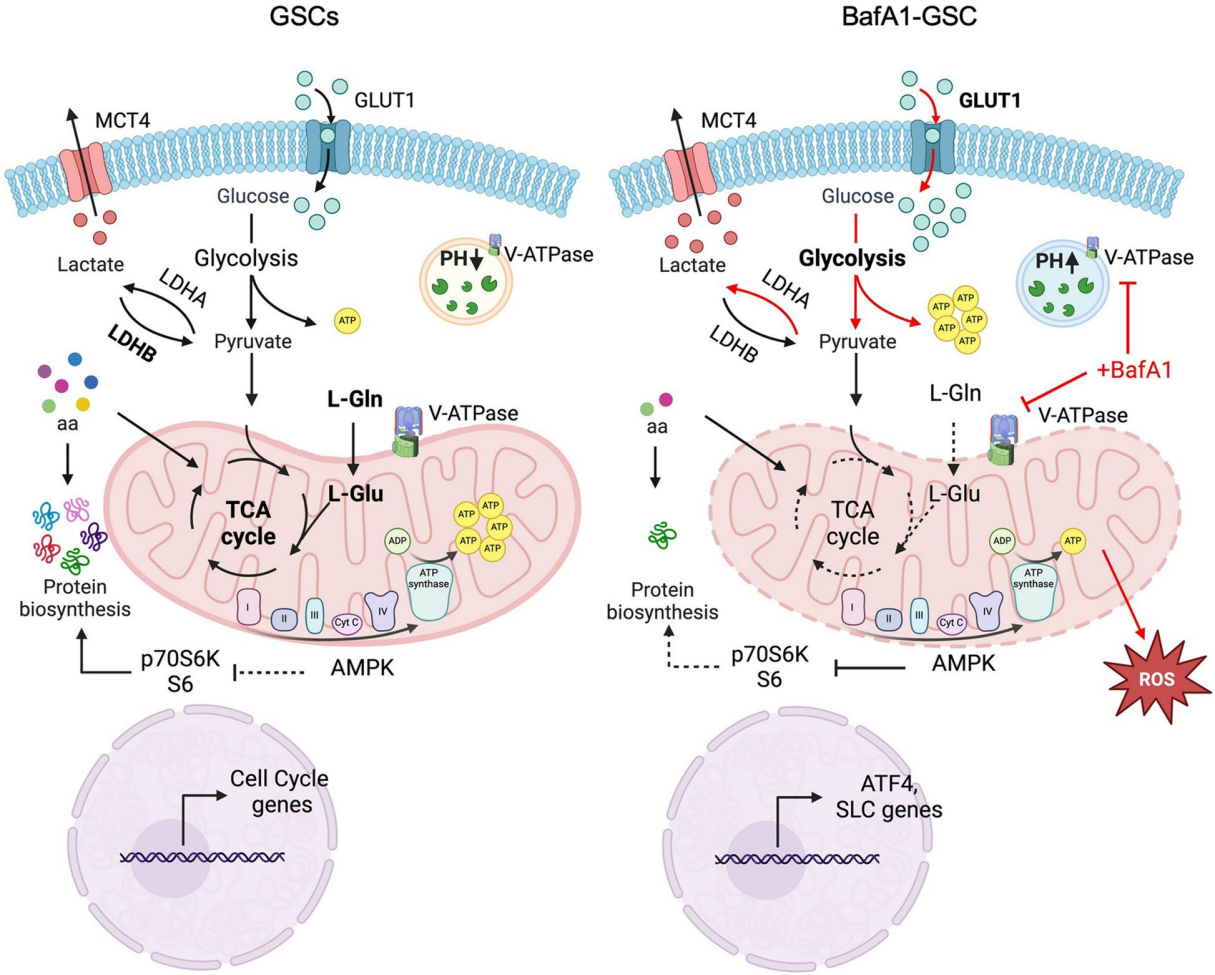
Schematic summary of V-ATPase-directed bioenergetic states in GSCs. GSCc are characterized by a mitochondrial pool of V-ATPase, and the active form of the pump supports GSCs growth and proliferation through mitochondria-dependent energetics

Finally, we measured the expression levels of the transcription factor ATF4, a nutrient sensor and master regulator of the amino acid deprivation response [35]. In BafA1 treated GSCs, the expression of ATF4 increased significantly (Fig. 4c). The combination of gene expression output and metabolomic analyses showed that BafA1 treatment promotes AMPK while inhibiting glutamate/glutamine metabolism (Fig. 4d and Suppl. Table S7). Accordingly, BafA1 treatment induces a significant impairment of protein biosynthesis in GSCs (Fig. 4e), in line with the reduced levels of amino acids observed in metabolomics (Fig. 5).

## Discussion

In this study, we present new evidence for a role of V-ATPase activity in the regulation of GSCs bioenergetic status required for tumor growth and invasion in vivo. We describe for the first time the localization of V-ATPase pump on GSCs mitochondria, besides the lysosomes. We demonstrated that V-ATPase targeting induces mitochondrial damage and metabolic rewiring, hijacking mitochondrial metabolism, protein synthesis and promoting ROS production. The higher glycolytic rate in BafA1-treated GSCs is accompanied by intracellular lactate accumulation, which is not exploited by GSCs for biosynthetic/survival purposes, in contrast to what has been previously described in normal astrocytes or neuroblastoma cells [32].

In GSCs BafA1 treatment specifically decreases the abundance of glutamine and glutamate, two amino acids that are crucial in maintaining growth and survival in GBM cells, including GSCs [31, 36].

V-ATPase-mediated bioenergetic rewiring, does not involve autophagy or mitophagy, confirming that these pathways are not exploited by glioma stem cells as salvage strategy in the event of a bioenergetic crisis [24]. This result is supported by the identification of a mitochondrial pool of V-ATPase, which affects organelle polarization, integrity and function. Similarly to previous evidence [8], V-ATPase acts as a sensor and transducer of amino acid availability, modulating mTORC1 signaling. In line with this, our results show that V-ATPase inhibition blunts phosphorylation (i.e. activation) of the S6-p70 kinase and of S6 ribosomal protein, two major effectors of mTORC1 signaling for biosynthetic functions accordingly, protein synthesis was significantly decreased in BafA1-treated GSCs. This cascade arrests GSC growth in vitro and in vivo.

Altogether our data suggest that targeting V-ATPase activity may be a novel potential vulnerability for glioblastoma. Inhibitors of V-ATPase activity may exert more efficacy when combined with metabolic targets, and this combinatorial treatment might deserve a future study, given that GBM is the most aggressive and most common form of primary brain tumor in adults. Within GBM, the presence of the GSCs niche supports rapid growth, infiltration into the surrounding brain tissue, and therapy resistance [3]. GSCs play a crucial role in disease recurrence after surgery and overall patients’ survival. In terms of bioenergetics, GSCs exhibit elevated metabolic plasticity, being able to adapt to and strive unfavorable conditions in terms of nutrients or oxygen availability and resist to chemo/radiotherapy regimens. GSCs heavily rely on glutaminolysis, showing a “glutamine addiction” phenotype, which involves the conversion of glutamine to glutamate in the mitochondria and subsequently to α-ketoglutarate, before entering the TCA cycle [31]. This supports both energy production and macromolecules biosynthesis. Moreover, GSCs show increased mitochondrial biogenesis to support their high energy demand. In the Drosophila preclinical model of brain tumor, which contains a rapidly dividing stem cell population, it has been shown that oxidative phosphorylation fueled by glutamine, rather than glucose, is required for tumor cell immortalization, and that its blockage arrests proliferation of those tumor progenitor cells and prevents tumorigenesis, suggesting that mitochondrion-dependent bioenergetics can be a key contributor of tumorigenesis [37, 38]. In contrast, self-renewal of normal brain progenitor cells is associated with aerobic glycolysis [39].

The importance of OxPhos in promoting and sustaining tumorigenicity has been also described in tumor stem cells from carcinomas of the pancreas [40], skin [41, 42], lung [43], and ovary [44].

In the context of patients-derived GSCs from primary tumors, Vlashi and collaborators showed that GSCs were less glycolytic comparted to differentiated glioma cells, displaying lower glucose uptake rates, lower lactate production, and higher ATP levels. Moreover, GSCs differentiation provoked a metabolic switch for ATP production from OxPhos to glycolysis [4].

Our study adds on this previous knowledge proposing the exploitation by GSCs of an active V-ATPase pump to support mitochondrial homeostasis, bioenergetics and growth.

## Conclusions

The inhibition of V-ATPase activity is sufficient to slow glioma growth and invasion in vivo, proposing the proton pump as a crucial factor for GSCs tumorigenicity. Our previous data indicated that GSCs with higher V-ATPase activity were more tumorigenic in vivo and in vitro, and that glioma patients with overexpression of the V-ATPase subunit G1 had a poorer prognosis irrespective of their grade and molecular characteristics [25]. Here we described the critical role of mitochondrial metabolism in mediating V-ATPase oncogenic function, and we proposed V-ATPase targeting as a new metabolic vulnerability to exploit in the clinical setting.

## Supplementary Information

Supplementary Material 1.

Supplementary Material 2.

## Data Availability

All data generated or analysed during this study are included in this published article and its supplementary information files.

## Abbreviations

GBM: Glioblastoma
GSC: Glioma stem cell
BafA1: Bafilomycin A1
ROS: Reactive oxygen species
TME: Tumor microenvironment
OxPhos: Oxydative phosphorylation
