# Supplementary material for: V-ATPase in glioma stem cells: a novel metabolic vulnerability
Source: J Exp Clin Cancer Res. 2025 Jan 17;44:17. doi: 10.1186/s13046-025-03280-3 (PMC11740391; doi:10.1186/s13046-025-03280-3)
Supplement: Supplementary file 1 — Supplementary Material 1. [file 13046_2025_3280_MOESM1_ESM.docx]

**V-ATPase in glioma stem cells: a novel metabolic vulnerability**

**SUPPLEMENTARY INFORMATION**

Supplementary Information includes Suppl. Methods, eight Suppl. Figures and seven Suppl. Tables (provided in separated spreadsheet)

**SUPPLEMENTARY METHODS**

Total and mitochondrial proteins isolation and quantification

Total protein extracts were collected incubating GSCs pellets with lysis buffer (Tris-HCl 50 mM, NaCl 137 mM, Triton X-100 1%, SDS 0,1% protease and phosphatase inhibitors; Roche) overnight at +4°. Then, samples were sonicated (5 cycles: 30 sec on, 30 sec off, 160W) using the Bioruptor (Diagenode) and cleared by centrifugation for 15 min at 15000 rcf at 4°C.

Mitochondria were isolated using the Mitochondria Isolation kit for cultured cells (Option B, ThermoFisher Scientific, cat. 89874). Briefly, GSCs were disaggregated by pipetting and 3-7x10^6^ cells were homogenized in reagent A by Dounce Tissue Grinder (Fisher Scientific) using 90 strokes to ensure efficient cell lysis. Cell homogenates were resuspended in reagent C and centrifuged twice at 700 rcf for 10 min 4°C. Supernatants were transferred and centrifuged at 2500 rcf for 15 min 4°C. The remaining pellet, which contains mitochondria, was washed with reagent C, then resuspended in TBS chaps-2% (28300, Thermo Fisher Scientific) and vortexed to obtain mitochondrial proteins. The reagents and TBS chaps-2% were supplemented with the protease inhibitor EDTA free (4693159001, Roche). Protein content was quantified by Micro BCA™ Protein Assay Kit (23235, Thermo Fisher Scientific).

Immunoblotting and protein array

For immunoblot, 30µg of total protein or 10 ug of mitochondria proteins were loaded on 12% acrylamide/bis-acrylamide gel (37:1, Sigma-Aldrich) and transferred on nitrocellulose membranes (BioRad), blocked with 5% skim powder milk (70166, Sigma-Aldrich). Membranes were incubated with the following primary antibodies: anti-LDHA, anti-LDHB, anti-GLUT1, anti-PDK1, anti-MCT4, anti-VDAC, anti-COX IV, anti-SDHA, anti-PDH, anti-CYC, anti-β-tubulin, anti-LC3, anti-SQSTM1, anti-phospho-mTor, anti-total mTor, anti-PACRG, anti-PINK1, anti-BNIP3L anti-FUNDC2. Anti-βActin and anti-vinculin were used as loading controls. Antibodies dilution and brands are shown in Supp. Table S1.

After primary antibody, membranes were incubated with the appropriate HRP-secondary antibody (1:3000, 170-6516 and 170-6515 BioRad) for 1h at room temperature.

For the phospho-MAPK-Array (ARY002B, R&D Systems), membranes were incubated with 400µg of total proteins and Detection Antibody Cocktail overnight at 4°C. The following day, membranes were incubated with Streptavidin-HRP. Signals were detected by ChemiDoc (Bio-Rad) and analyzed using Fiji ImageJ software (<https://imagej.net>).

Standard, calibration and quality control solutions

For the preparation of internal standard solution (IS), D-glucose-13C6 and L-lactate-13C3 were purchased from Sigma-Aldrich (≥ 99% atom 13C; Milan, Italy). For the mobile phases, standard solutions, assay optimization and samples preparation, methanol (MeOH), acetonitrile (CH3CN), and formic acid (all LC-MS/MS grade, Sigma-Aldrich, Milan, Italy) were used. Purified water was obtained using a Milli-Q Plus ultra-pure water system (Millipore, Milford, MA). Aqueous standard solutions containing both GLU and LACT at concentrations of 500, and 5 mg/L were prepared. An IS solution containing the isotopically labelled analytes, each at the concentration of 500 mg/L, was prepared in water. Standard and IS solutions were stored at –20 °C in the dark in plastic tubes. Under these conditions, the solutions were stable up to 6 months.

Calibration solutions containing the analytes, each at the concentrations of 0.025, 0.05, 0.5, 1.0, 10, 100, 500 mg/L, and QC solutions at 0.1, 5.0 and 50 µg/L, for low- medium- and high-QC, respectively, were prepared by adding suitable amounts of standard solutions to water.

Before starting the analytical procedure, the IS solution was added to each calibration solution, QC solution and unknown sample to the final concentration of 5 mg/L.

LC-MS/MS analysis

The LC separation was performed with the chromatographic column kept at room temperature using a linear gradient obtained with the A phase, 0.1% formic acid in water, and the B phase, 0.1% formic acid in CH3CN, flowing at 300 µL/min. The gradient was programmed as follows: 0 - 2 min, 80% B isocratic; 2 – 5.5 min, from 80% to 5% B; 5.5 – 6.5 min, 5% B isocratic; 6.5 - 7 min, from 5% to 80% B; 7 - 12 min, 80% B isocratic. The mass spectrometer was operated using the H-ESI in the negative ion mode. The ionization source parameters were: spray voltage 4,000 V; ion transfer tube temperature 227 °C; vaporization temperature 293 °C; nitrogen as sheath and auxiliary gas operating at the pressure of 30 and 15 units (arbitrary scale); tube lens offset 57 V. Collision-induced dissociation was performed using Ar as collision gas at a pressure of 1.5 mTorr in the collision cell. Quantification was based on multiple reaction monitoring following the transitions: m/z 179→43 + 179→89 (collision energy 25 and 12 eV, respectively) for GLU; m/z 185→45 + 185→92 (collision energy 25 and 12 eV, respectively) for GLU-13C6; m/z 89→43 + 89→71 (collision energy 13 eV) for LACT; m/z 91→45 + 91→74 (collision energy 14 and 13 eV, respectively) for LACT-13C3. The retention times were 3.86 min and 3.84 min for glucose e lactate, respectively.

**SUPPLEMENTARY FIGURES**


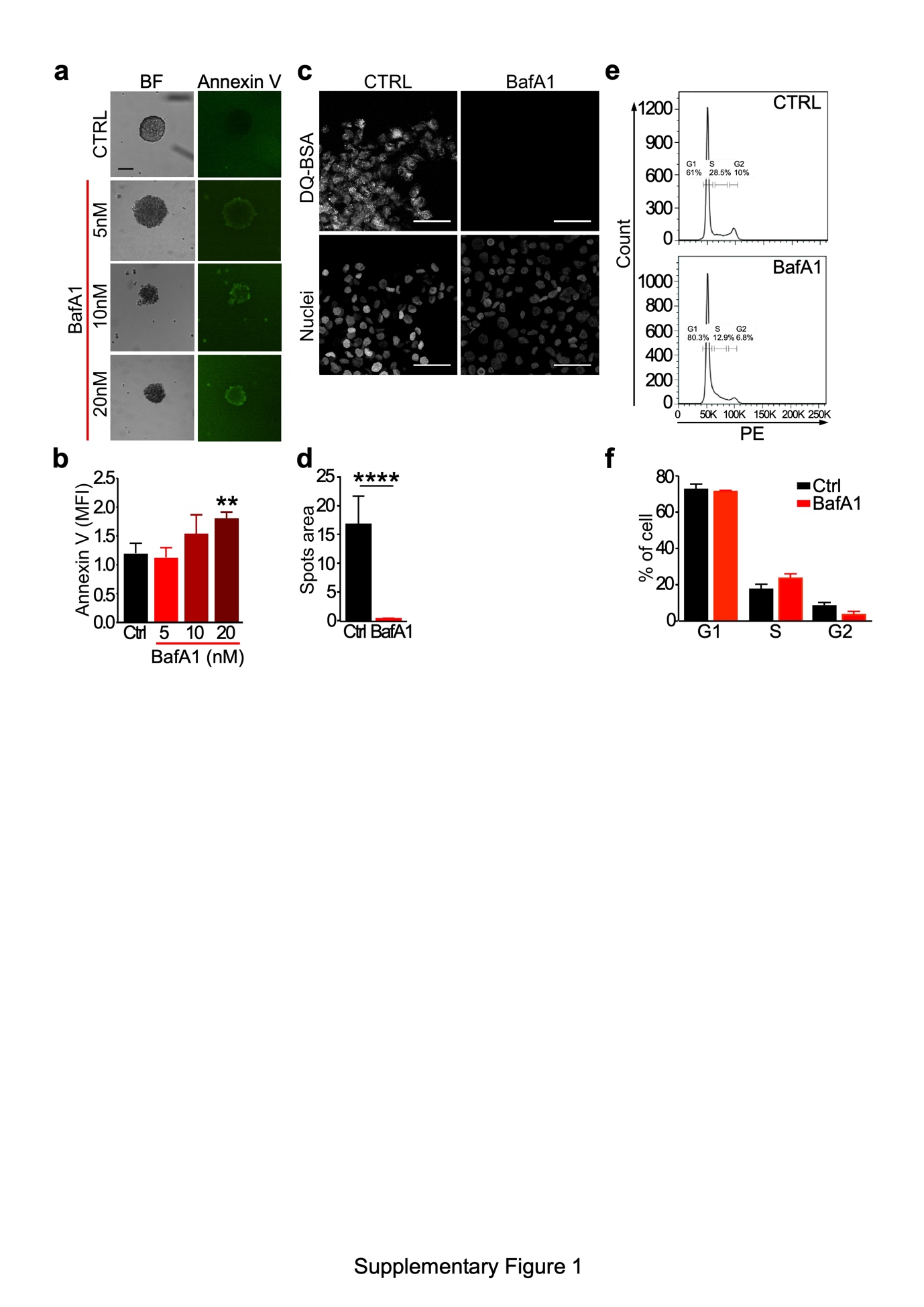


**Supplementary Fig. 1. BafA1 decreases lysosomal acidification and activity and induces cell cycle arrest in GSC.**

**a,b**) GSCs were treated with vehicle (Ctrl) or with the indicated doses of Bafilomycin A1 (BafA1) and cell death was monitored after 24h using AnnexinV staining. Images were acquired using a time-lapse microscope at 5x of magnification (**a**), and staining intensity was quantified (MFI, mean fluorescence intensity; **b**). Scale bar: 100μm. **, p=0.008 by Mann-Whitney U test. Bars, mean with SEM. **c,d**) GSCs were incubated with vehicle (Ctrl) or 5nM of BafA1 for 24h after which the proteolytic ability of GSCs was measured using the DQ-BSA assay. Images were acquired by a confocal Microscope at 63x of magnification (**c**) and analyzed by Fiji ImageJ software (**d**). Scale bar 50µm. ****, p<0.0001 by Mann-Whitney U test. Bars, mean with SEM. **e,f**) Cell cycle was measured after 48h of BafA1 5nM treatment and percentage of positive cells were quantified by FlowJo software. Representative images are shown for **a**, **c**, and **e** panels.


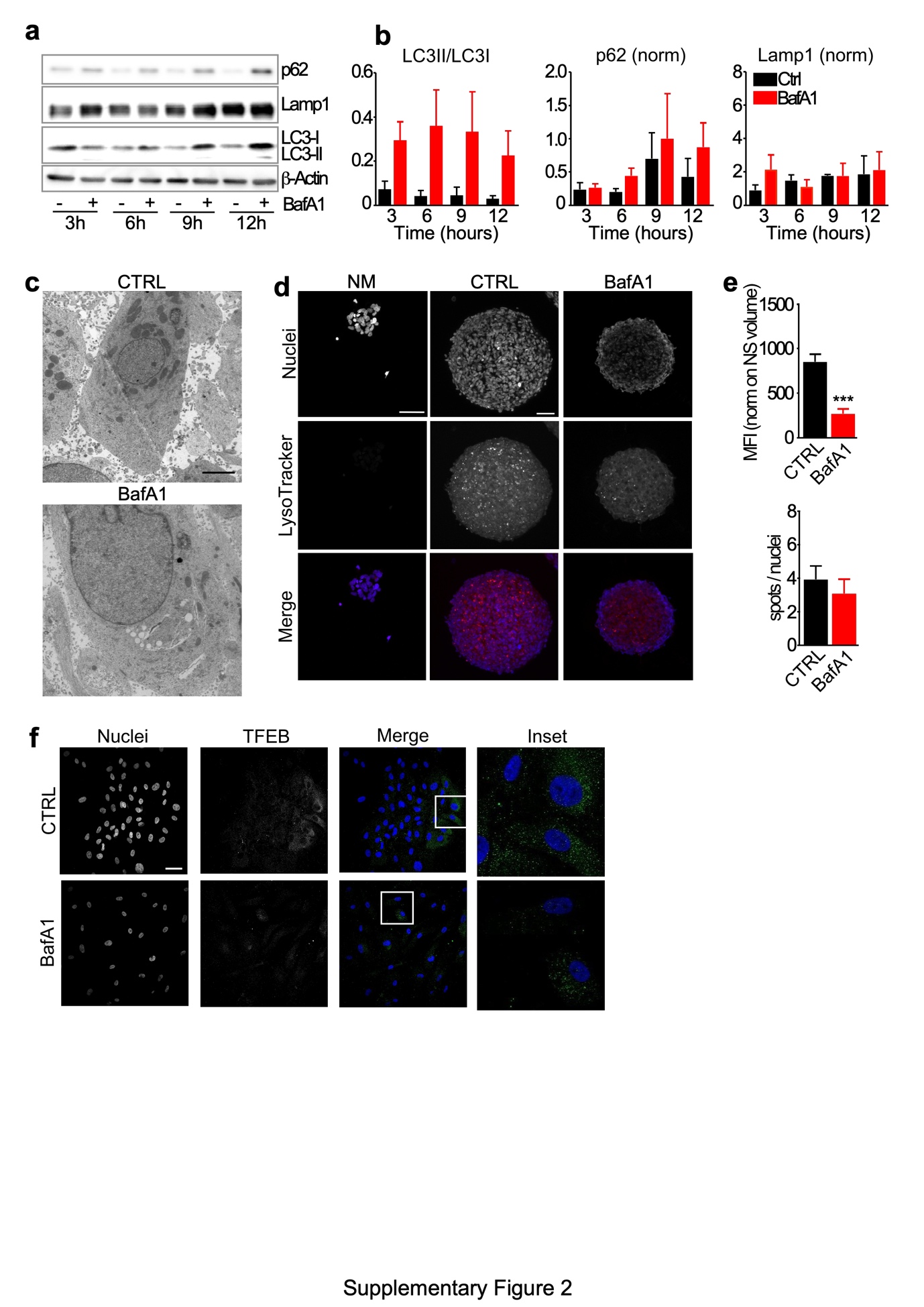


**Supplementary Fig. 2 Autophagy is not crucial for fueling GSCs bioenergetics.**

(**a,b**) Expression of LC3B-II, p62 and Lamp1 were analyzed by western blot in GSC treated or not with BafA1 at the indicated time points. **c**) The presence of autophagosome or autophagolysosomes was assessed by electron microscopy in GSC treated with vehicle or BafA1; representative images are shown. Scale bar, 2 μm **d,e**) Lysosomes were stained with LysoTracker dye and their number (positive spots) and intensity (MFI) was quantified using Volocity software. Representative images are shown (**d**); NM, not marked; Bars, mean with SEM; ***p=0.002 by Mann-Whitney U test. **f**) Nuclear TFEB expression was analyzed by immunofluorescence in CTRL and BafA1 treated cells GSC.


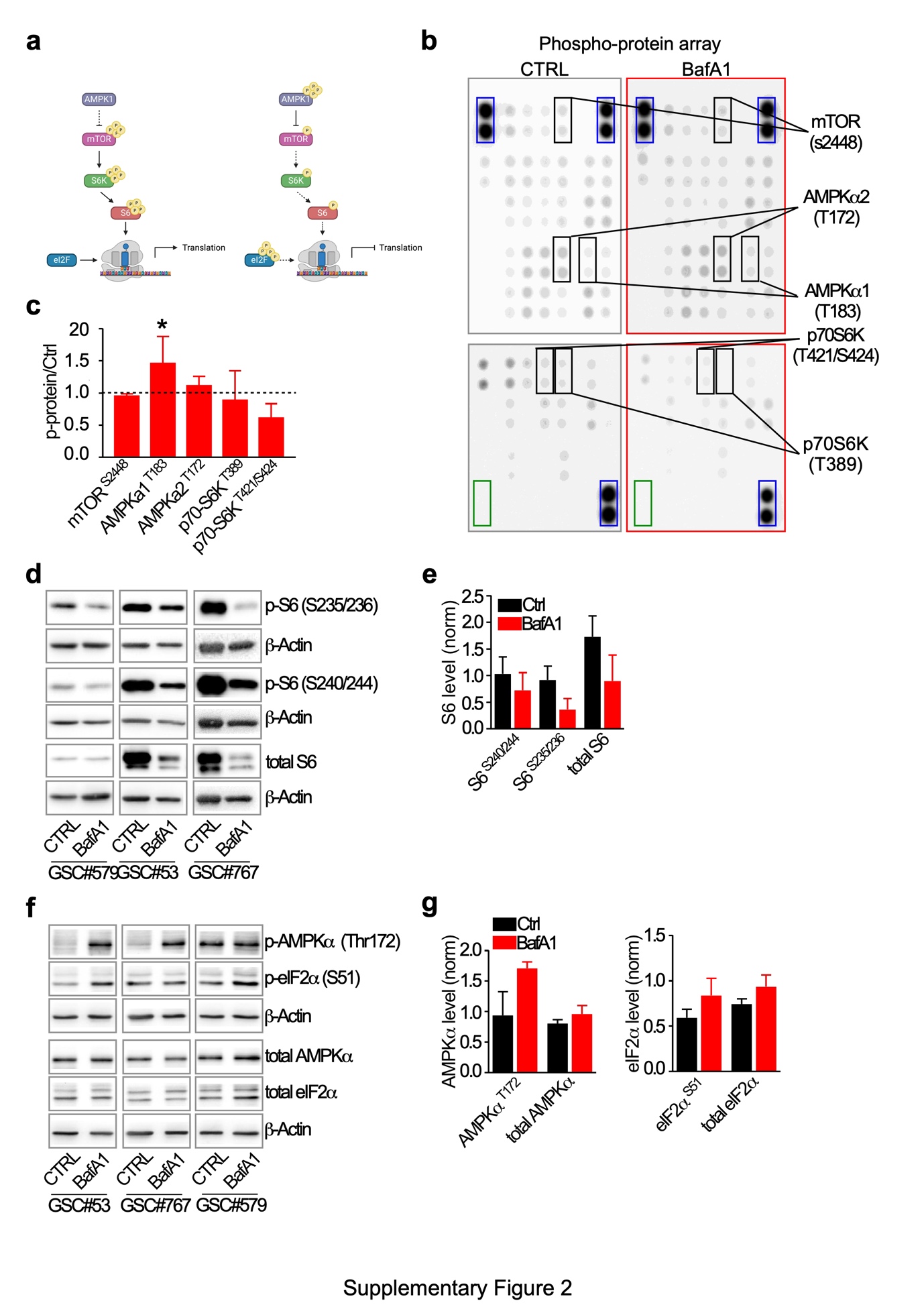


**Supplementary Fig. 3 The block of V-ATPase activity represses the mTOR pathway**

**a**) Schematic of the AMPK/mTOR signaling. Phosphorylation levels of mTOR and its downstream (p70S6K, S6, and eIF2α), and upstream (AMPKa) proteins were evaluated by a MAPK phospho-array (**b,c**) and by western blot (**d-g**). The array positive and negative control spots are indicated by blue and orange rectangles, respectively (**b**). Densitometric analysis was performed by Fiji ImageJ software (**c, e** and **g**). *, p=0.04 by Mann-Whitney U test. Bars, mean with SEM.

**
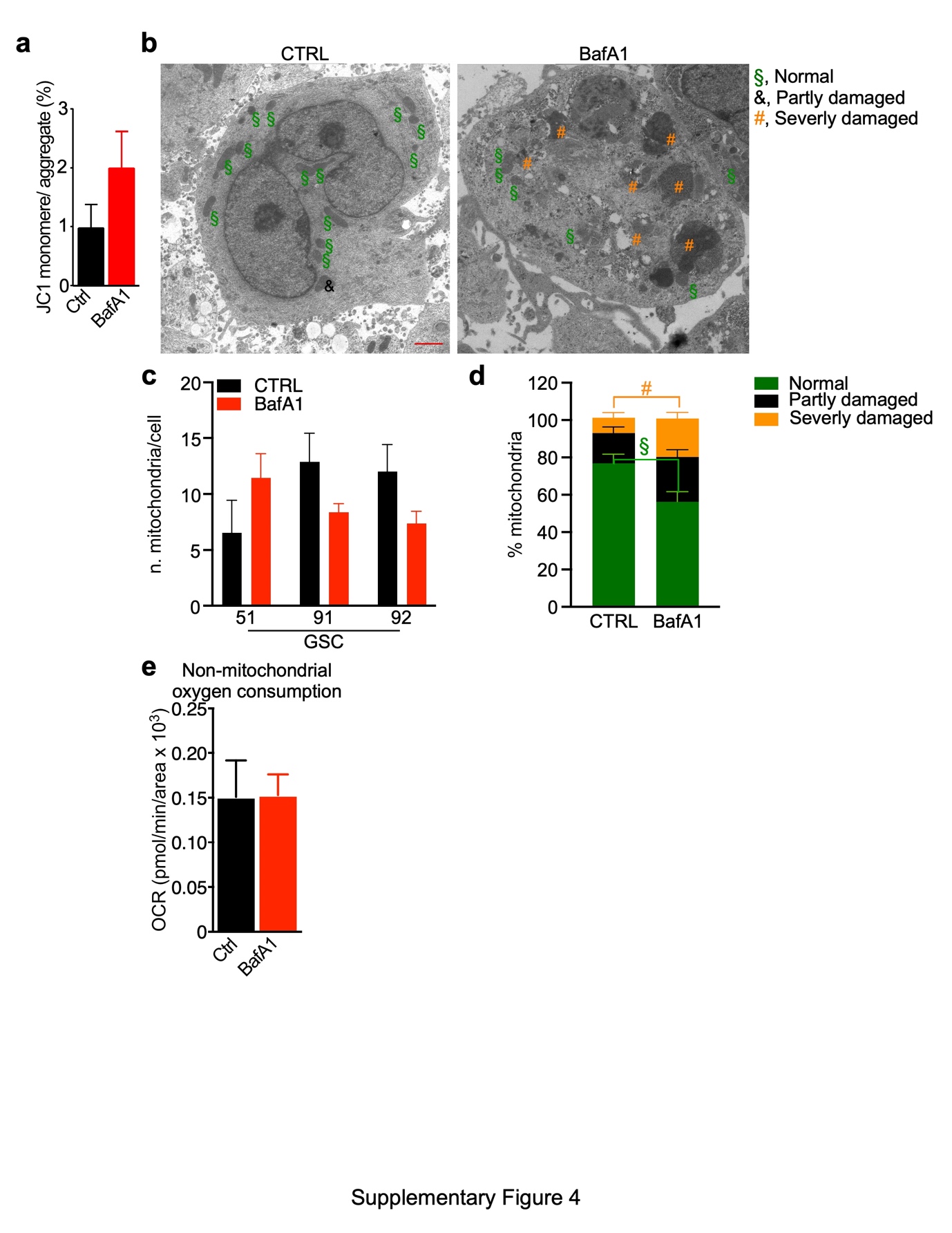
**

**Supplementary Fig. 4. Bafilomycin A1 treatment damages GSC mitochondria**

Mitochondria dysfunction was assessed by JC1 assay after 48h of 5nM-BafA1 treatment using FACS CANTO (**a**). Total, partly damaged and severely damaged mitochondria were scored in electron microscopy images (**b-d**) in GSC treated with vehicle (CTRL) or 5nM-BafA1. ^#^, p=0.001; ^§^, p=0.005 by Mann-Whitney U test. The non-mitochondria oxygen consumption rate (OCR) was evaluated using the Seahorse Mito stress test kit (**e**) in the indicated conditions.


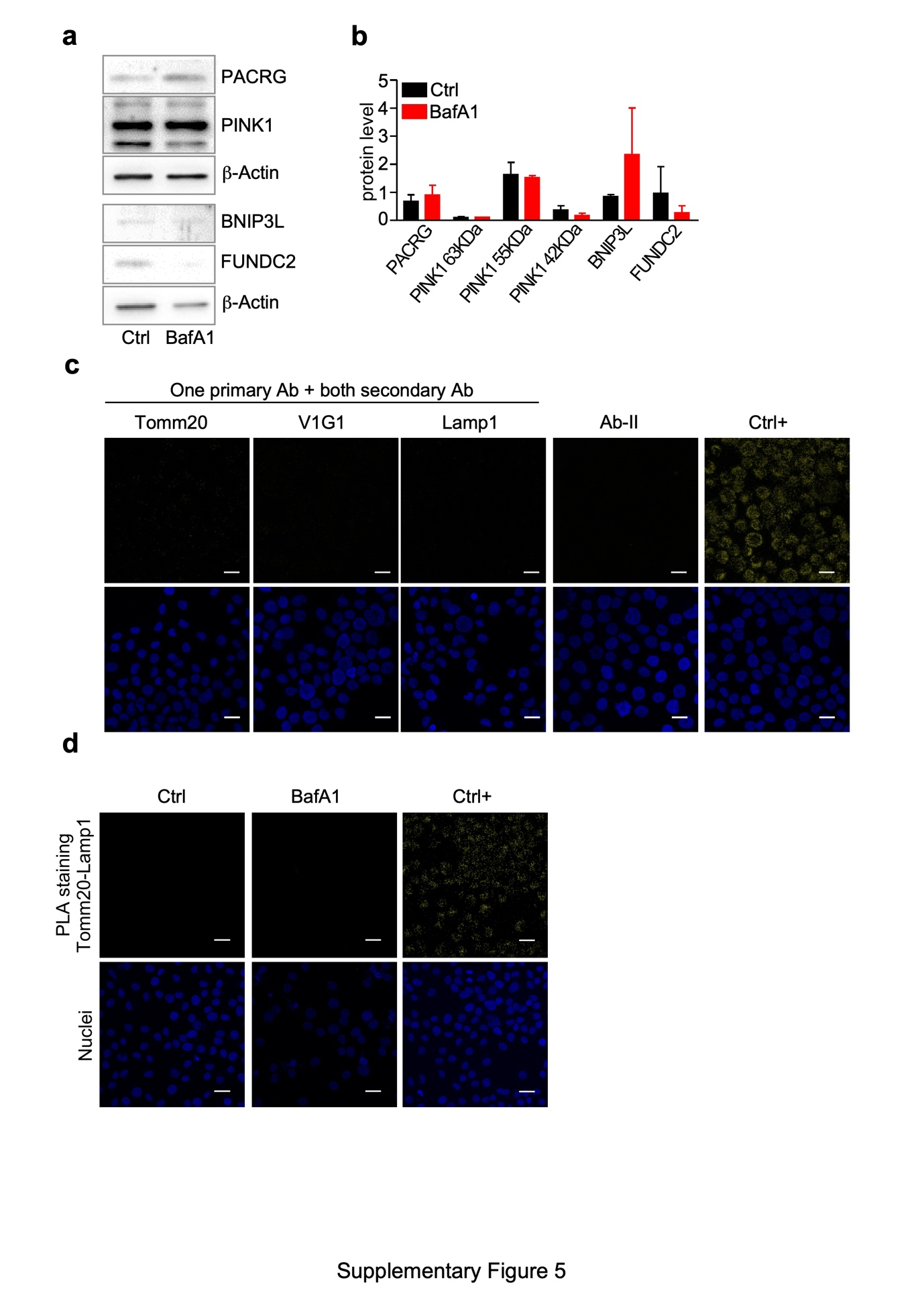


**Supplementary Fig. 5 V-ATPase G1 is present on GSC mitochondria, and its inhibition does not modulate mitophagy**

GSC were treated for 48h with 5nM-BafA1 and expression of mitophagy protein markers (PACRG, PINK1, BNIP3L, FUNDC2) was evaluate by western blot (**a**). Densitometric analysis was performed by Fiji ImageJ software (**b**). Bars, mean with SEM.

Negative controls for PLA were represented by single primary antibody staining (first 3 panel from the left: Tomm20, V1G1 and Lamp1) and without both primary antibodies (4^th^ panel, AbII). As positive CTRL two Tomm20 antibodies were used (first panel from the right). (**c**) Images were acquired by Leica SP8 Confocal Microscope (Leica Microsystems, 63x of magnification). Scale bar 20µm.

Colocalization between mitochondria and lysosomes (Tomm20-Lamp1) were evaluated by PLA (**d**). Images were acquired by Leica SP8 Confocal Microscope (Leica Microsystems, 63x of magnification). Scale bar 20µm.

**Supplementary Fig. 6. Bafilomycin A1 treatment in GSCs decreases the expression of mitochondria biogenesis and cell cycle genes**

Gene expression analysis of transcripts involved in mitochondrial biogenesis (**a**) and cell proliferation (**b**). Data are presented as violin plot, and each case is a dot. §, p=0.001; #, p=0.005; *, p=0.01; **, p=0.007; ***, p=0.002 by Mann-Whitney U test

**
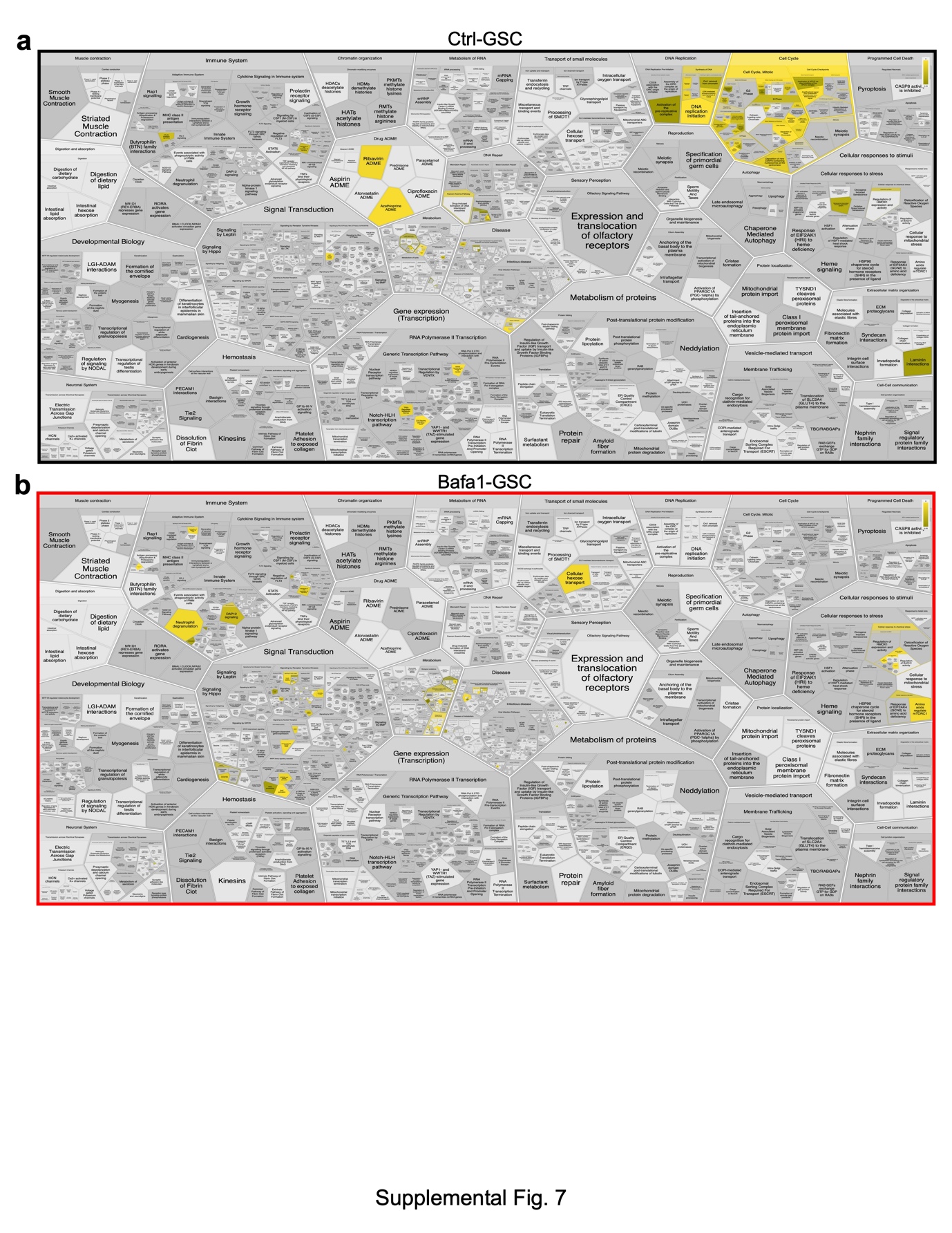
**

**Supplementary Fig. 7 Pathways analysis in control or BafA1-treated GSC**

A metabolic gene expression panel (n=748 genes; see also Suppl. Table S4) was analyzed in GSCs treated with vehicle (Ctrl) or 5nM Bafa1 for 48h. List of genes whose expression was significantly up (adj p value < 0.05 and log2FC> 0.5) in Ctrl-GSCs or in BafA1-GSCs were separately imported in Reactome web-tool and the pathway analysis was performed with Voronoi pathway visualization.

**
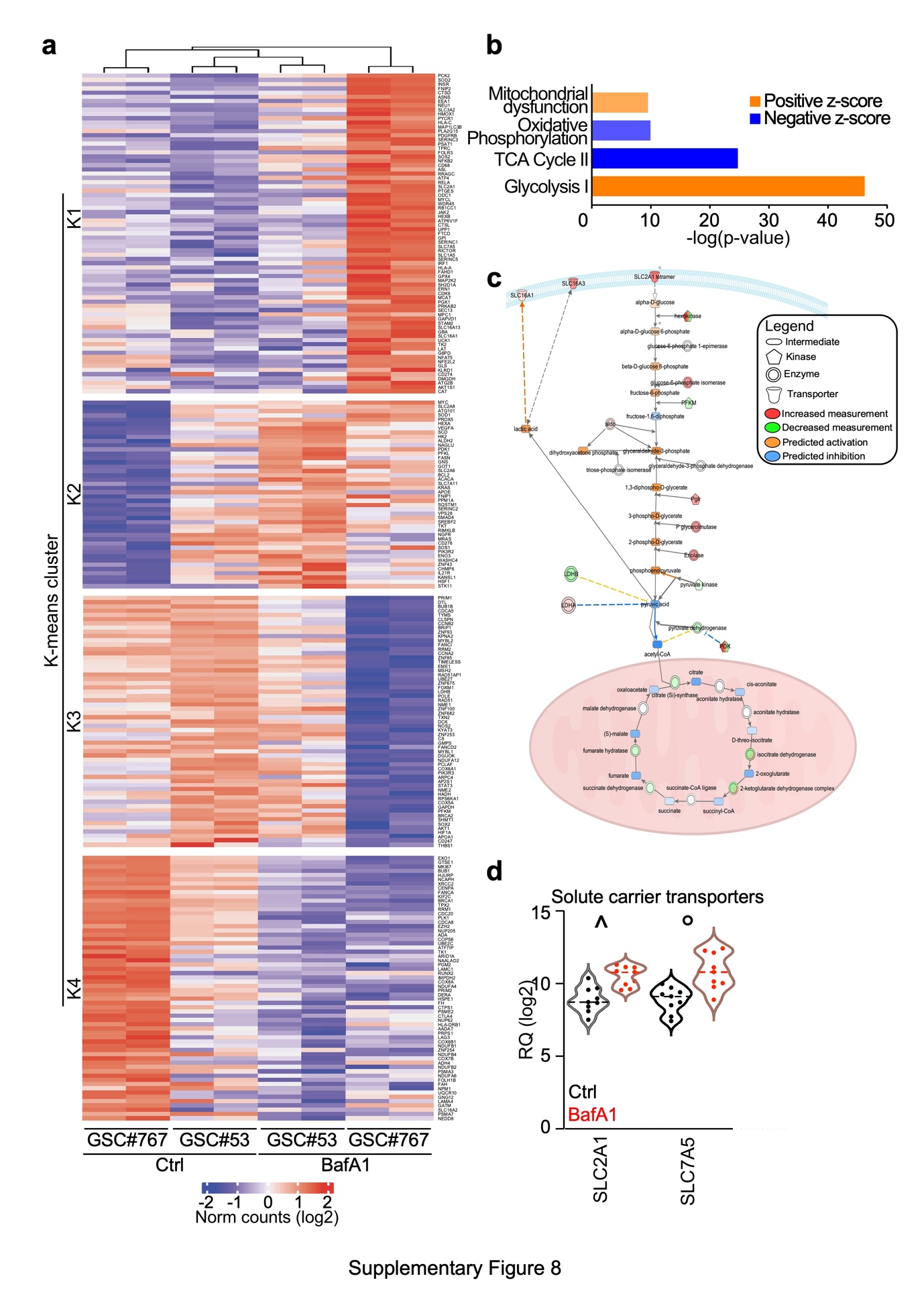
**

**Supplemental Fig. 8 Bafilomycin A1 treatment upregulates genes involved in glycolysis**

Differential expression analysis of metabolic genes in GSC treated with vehicle or 5nM-BafA1. The heatmap shows k-means clusters of genes (**a**). Ingenuity pathway analysis (**b,c**) shows prediction of active (positive z-score, orange) or repressed (negative z-score, blue) signaling and genes.

The expression of the glucose transporter (SLC2A1) and of the L-type amino acids (SLC7A5) was significantly decreased in BafA1-treated GSC (d). ^, p=0.04; °, p=0.02 by Mann-Whitney U test. Data are presented as violin plots where each dot is a sample.
